# Therapeutic Plasma Exchange in Refractory Atrial Fibrillation Secondary to a Thyroid Storm

**DOI:** 10.7759/cureus.49109

**Published:** 2023-11-20

**Authors:** Sara AlShehri, Marwah Bafadel, Naji Aljohani

**Affiliations:** 1 Endocrinology, Diabetes, and Metabolism, Princess Nourah Bint Abdulrahman University, Riyadh, SAU; 2 Endocrinology and Diabetes, King Fahad Medical City, Riyadh, SAU; 3 Obesity, Endocrine, and Metabolism Centre, King Fahad Medical City, Riyadh, SAU; 4 College of Medicine, Alfaisal University, Riyadh, SAU

**Keywords:** graves’ disease, apheresis, plasma exchange, atrial fibrillation, thyroid storm

## Abstract

Thyrotoxic crisis (thyroid storm) is a severe form of hyperthyroidism. It has a wide range of symptoms, including cardiovascular manifestations that can be life-threatening and require prompt management. Cardiac manifestations of thyroid storm include hypertension, tachycardia, congestive heart failure, cardiac ischemia, and atrial fibrillation. Standard therapeutic approaches of thyroid storm are based on the use of thionamides, corticosteroids, and nonselective beta-blockers to inhibit thyroid hormone synthesis and release while also blocking thyroxin's peripheral effects. This approach is an effective measure in the management of these manifestations. However, when a patient fails to respond to conventional therapy, or there is end organ damage and surgery is not feasible, therapeutic plasma exchange (TPE) is a life-saving measure. Here, we report a case of a 27-year-old female who is known to have hyperthyroidism, but she was not compliant with her medication. She presented with symptoms of hyperthyroidism and was found to have a thyroid storm with refractory atrial fibrillation. Despite the effort to control her symptoms, she developed cardiac arrest, and after 15 minutes of cardiopulmonary resuscitation, she was revived. Amiodarone, beta-blockers, and cardioversion failed to improve the patient's status, and her condition deteriorated. So, we decided to use TPE, and she received three sessions in total. After the first session of TPE, her rhythm converted to sinus rhythm, and her heart rate was controlled to less than 100 beats per minute. She showed dramatic improvement clinically and biochemically. In conclusion, cardiac complications are potentially lethal complications of thyroid storm. Prompt restoration of a normal thyroid state can reverse these complications. When conventional therapy fails to ameliorate symptoms or organ deterioration is rapid and severe, TPE can be a safe and effective measure.

## Introduction

A thyrotoxic crisis (thyroid storm) is a rare severe manifestation of thyrotoxicosis, which can present with multiorgan failure secondary to a hypermetabolic state. It is often precipitated by multiple acute conditions, such as thyroid or nonthyroidal surgery, trauma, or infection. Despite extensive medical therapies, there is a 10-30% death rate from a thyroid storm. It is an uncommon but deadly illness [1].

The first-line treatment is based on blocking the production of thyroid hormone and decreasing the peripheral conversion of thyroxine (T4) to triiodothyronine (T3) by thioamides; also, beta blockers can be added to control the heart rate, glucocorticoids to reduce the T4 to T3 conversion, and iodine solutions (SSKI® or Lugol’s solution) to decrease thyroid hormone level and reduce blood flow within thyroid gland to block the release of the hormones. Bile acid sequestrants (cholestyramine) can be used, which act by decreasing the enterohepatic recycling of thyroid hormones [2].

This case reports the use of therapeutic plasma exchange (TPE) in a patient who has persistent atrial fibrillation (A.fib) secondary to a thyroid storm. She was treated with three sessions of plasmapheresis, which showed significant improvement in her symptoms. The reporting of this case describes the importance of the utility of plasma exchange in the management of refractory A.fib secondary to thyroid storm.

This case report was previously presented as a meeting poster at the Endocrine Society Meeting (ENDO2023) on July 17, 2023.

## Case presentation

A 27-year-old female, known to have Graves’ disease (GD) diagnosed at the age of 18 years, presented to the emergency department with a history of palpitations, dizziness, shortness of breath, lower limb swelling, fever, and diarrhea for two days. Her vital signs showed a heart rate of 190 beats/minute, blood pressure of 82/47 mmHg, temperature of 38.5°C (101.3°F), and respiratory rate of 22 breaths/minute. Upon examination, the thyroid gland was diffusely enlarged with bilateral orbitopathy.

**Figure 1:**
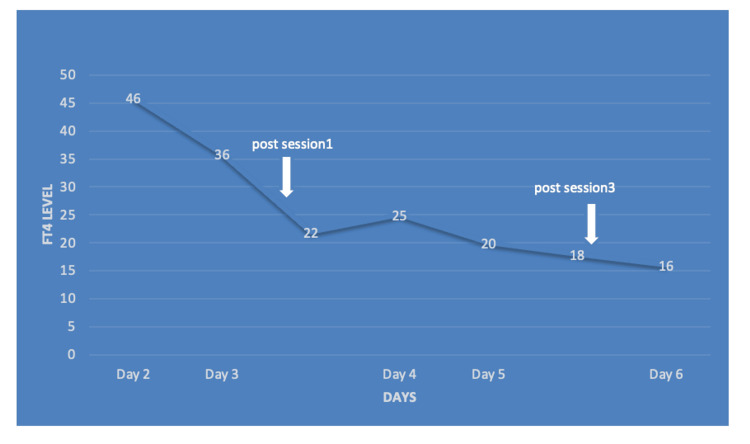
Free T4 level throughout the patient’s course revealed a substantial decrease with TPE sessions. TPE: therapeutic plasma exchange.

In regards to her GD, she had previously medically managed with methimazole but had not been compliant with it for the past year. She had a history of miscarriage two weeks back. Her free thyroxine (FT4) was > 64.35 pmol/l (normal range: 9.00-19.00 pmol/l), free triiodothyronine (FT3) was >30.72 pmol/l (normal range: 5.800-14.350 pmol/l), and thyroid-stimulating hormone (TSH) was undetectable (<0.001) at presentation. Thyroid storm diagnosis was established based on her findings and according to the Burch-Wartofsky Point Scale for the diagnosis of thyroid storm, on which she scored 80 (Table 1). She was initially managed with intravenous fluids, 200 mg propylthiouracil (PTU) every four hours, 60 mg propranolol every six hours, 100 mg hydrocortisone every eight hours, 250 mg potassium iodide (SSKI®) four times a day, and 300 mg cholestyramine four times a day. Eight hours later, she developed unstable A.fib and ended up with cardiac arrest, which required resuscitation for 15 minutes. Despite extensive medical treatment, the patient's condition was not improved despite a decreased FT4 level. She still had persistent unstable A.fib even after she received multiple shocks and the cardiology team started her on amiodarone but she did not respond to it, so it was stopped. Her condition was further complicated by elevated liver enzymes and anuric acute kidney injury. Continuous renal replacement therapy (CRRT) was started. Based on that, we decided to use TPE, and her first session was on the third day of admission. She received a total of three sessions. During the first TPE session, 3 L of plasma was extracted over two hours and replaced with intravenous 5% albumin and fresh frozen plasma (1.5:1.5 ratio). She tolerated TPE well with dramatically decreased FT4 with each session, as shown in Table 2 and Figure 1. On day 15, she underwent a total thyroidectomy with tracheostomy without complication. The patient's condition became more stable vitally and metabolically, so they transferred her to the general medical ward to complete her management and follow-up by the endocrine team. After a couple of weeks, the patient unfortunately developed septic shock and she passed away.

**Table 1:** Burch-Wartofsky Point Scale (BWPS) for thyrotoxicosis. Total score >45 = thyroid storm, 25-45 = impending thyroid storm, and <25 = storm unlikely.

Criteria	Points
Thermoregulatory dysfunction (temperature)	
99.0-99.95	0
100.0-100.9	10
101.0-101.9	15
102.0-102.9	20
103.0-103.9	25
>104.0	30
Cardiovascular	
Tachycardia, beats per minute	
100-109	5
110-119	10
120-129	15
130-139	20
>140	25
Atrial fibrillation	
Absent	0
Present	10
Congestive heart failure	
Absent	0
Mild	5
Moderate	10
Severe	20
Gastrointestinal-hepatic dysfunction manifestation	
Absent	0
Moderate (diarrhea, abdominal pain, nausea/vomiting)	10
Severe (jaundice)	15
Central nervous system disturbance	
Absent	0
Mild (agitation)	10
Moderate (delirium, psychosis, extreme lethargy)	20
Severe (seizure, coma)	30
Precipitant history status	
Positive	0
Negative	10

**Table 2:** An overview of the course of treatment and T4 level with each TPE session. TPE: therapeutic plasma exchange; AKI: acute kidney injury; LFT: liver function test; DIC: disseminated intravascular coagulation; CRRT: continuous renal replacement therapy; HR: heart rate; PTU: propylthiouracil; #1: first session of TPE; #2: second session of TPE; #3: third session of TPE.

Day	T4 (normal range: 9-19)	T3 (normal range: 5-14)	Clinical
Day 2	46	30	PTU 300 mg every 4 hours, AKI, DIC, high LFT
Day 3 #1	36, post session = 22	17	3 inotropic support/HR: 190-200, shifted to carbimazole 30 mg every 4 hours.
Day 4 #2	25		Weaned off ionotropic support/HR: 140s/LFTs improved/CRRT started/amiodarone stopped
Day 5 #3	20, post session = 18	5	HR: 120s/LFT improved/carbimazole 30 mg every 6 hours
Day 6	16	3.7	HR: 80-90/amiodarone oral

## Discussion

A thyroid storm is a life-threatening endocrine emergency that can be untreated or incompletely treated hyperthyroidism precipitated by infection, surgery, or trauma [3]. Our patient's trigger was her recent history of miscarriage (15 days from her presentation) in addition to medication noncompliance. These led to her acute cardiac symptoms and severe cardiac dysfunction with left ventricular ejection fraction (LVEF) of 25% on transthoracic echocardiogram (TTE). While no validated clinical tools exist to diagnose thyroid storms, the Burch-Wartofsky Point Scale is commonly used [4]. In this case, the patient's score was more than 80, indicating a thyroid storm that requires immediate aggressive treatment.

The effects of hyperthyroidism on cardiovascular hemodynamics can exacerbate pre-existing cardiac disease or may cause cardiac complications in individuals with structurally normal hearts [5]. As a result of increasing heart rate, myocardial contractility, and oxygen consumption, hyperthyroidism can unmask numerous cardiovascular complications, including silent coronary artery disease and compensated heart failure, which can be life-threatening in an acute thyroid storm. A.fib is the most common cardiac complication associated with hyperthyroidism, affecting up to 15% of patients [5]. Restoration of the euthyroid state can reverse these cardiovascular changes in almost all cases [6].

The first step in managing A.fib in the setting of hyperthyroidism is to control the ventricular response with the use of beta blockers [6]. In this case, the patient was started on beta blockers despite her severely reduced ejection fraction, she tolerated it but she was not controlled. Amiodarone is an iodine-rich antiarrhythmic drug, which was shown to be effective in treating patients with ventricular and atrial arrhythmias. The thyrotoxic effect of amiodarone can be prevented by using amiodarone in conjunction with antithyroid agents [7]. Our patient was started on amiodarone; however, A.fib was not controlled. Digoxin can be considered in thyrotoxic A.fib, but in hyperthyroid states, it requires higher doses due to decreased sensitivity of the hyperthyroid heart to it, also it has less predictable results [6]. In our patient, the renal function was deteriorating, which precluded digoxin use.

TPE is an effective measure for the treatment of severe thyroid storm that is complicated by cardiac or neurological dysfunction. Thyroid storm has been classified as a category II indication by the American Society for Apheresis (ASFA) [8]. It can provide rapid resolution of symptoms by removing T3 and T4 that is bound to albumin, autoantibodies, catecholamines, and cytokines. Also, providing unbound albumin to the patient will create a free binding site for the free thyroid hormone, which will further decrease thyroid hormone levels. It usually takes three to six procedures to achieve clinical stabilization with TPE, as in our patient’s case, who required three sessions to achieve clinical and biochemical improvement.

## Conclusions

In conclusion, cardiac complication is a potentially lethal complication of thyroid storm. Prompt restoration of a normal thyroid state can reverse these complications. When conventional therapy fails to ameliorate symptoms or organ deterioration is rapid and severe, TPE serves as a valuable adjunctive therapy. Rapidly removing circulating thyroid hormones and autoantibodies helps to attenuate the hypermetabolic state and stabilize the patient's condition. Our patient developed a thyroid storm that was complicated by refractory atrial fibrillation. Despite the limitations of data, TPE has successfully reversed her severe cardiac dysfunction, providing a rescue treatment.
